# Spatially Resolved Free Fatty Acid Profiling Reveals Region- and Age-Dependent Remodeling of Alzheimer’s Disease Plaques

**DOI:** 10.1021/jacsau.6c00649

**Published:** 2026-07-11

**Authors:** Sona Hakhverdyan, Sofie Hansson, Pascal Kadej, Sophia B. Orlovsky, Siarhei Hladkou, Birger Viirman, Anna Nilsson, Martin Lord, Stephanie M. Cologna, Justin T. Mohr, Stina Syvänen, Per E. Andrén, Wojciech Michno

**Affiliations:** † Department of Public Health and Caring Sciences, Molecular Geriatrics, 8097 Uppsala University, SE-75237 Uppsala, Sweden; ‡ Department of Chemistry, 14681 University of Illinois Chicago, Chicago, Illinois 60607, United States; § Department of Pharmaceutical Biosciences, Spatial Mass Spectrometry, Science for Life Laboratory, Uppsala University, SE-75123 Uppsala, Sweden; ∥ Department of Pharmacy, Science for Life Laboratory, Uppsala University, SE-75123 Uppsala, Sweden; ⊥ Laboratory of Integrative Neuroscience, University of Illinois Chicago, Chicago, Illinois 60607, United States; # Science for Life Laboratory, Uppsala University, SE-752 37 Uppsala, Sweden

**Keywords:** mass spectrometry imaging (MSI), matrix-assisted laser desorption/ionization (MALDI), Alzheimer’s disease (AD), free fatty acids (FFA), spatial lipidomics, lipid metabolism, amyloid-β (Aβ) pathology

## Abstract

Free fatty acids (FFAs) are bioactive mediators of inflammation, energy metabolism, and membrane remodeling, yet their spatial organization within the Alzheimer’s disease (AD) brain and at individual amyloid-β (Aβ) plaques has remained inaccessible. We developed a novel, chemically tailored MALDI workflow that enables simultaneous, spatially resolved detection of nearly 30 FFAs alongside over 100 complex lipid species within the same tissue section. Applying this approach to a transgenic AD mouse model across brain regions and disease stages, and combining it with single-plaque microenvironment analysis (SPMA) that treats each plaque as an individual analytical object, we uncover two previously inaccessible dimensions of plaque-associated lipid biology. FFA distributions form highly structured spatial compartments reflecting regional cytoarchitecture, with distinct enrichment of saturated, monounsaturated, and polyunsaturated species across cortical layers. Within Aβ plaques, nearly 75% of detected FFAs are significantly remodeled, with reciprocal enrichment of short saturated and highly unsaturated species alongside depletion of long-chain monounsaturated FFAs. This pattern is consistent with concurrent disruption of ELOVL-mediated elongation and FADS-mediated desaturation, including opposing enrichment of pro-inflammatory arachidonic acid and pro-resolving docosahexaenoic acid. Machine learning of single-plaque profiles reveals that FFA composition alone classifies plaque age with high accuracy, demonstrating that lipid remodeling continues after Aβ peptide composition has stabilized. Together, these findings establish spatial FFA profiling as a new analytical dimension in neurodegeneration research, revealing that Aβ plaques are dynamic lipid-metabolic microenvironments that continue to remodel long after Aβ deposition has stabilized.

## Graphical abstract

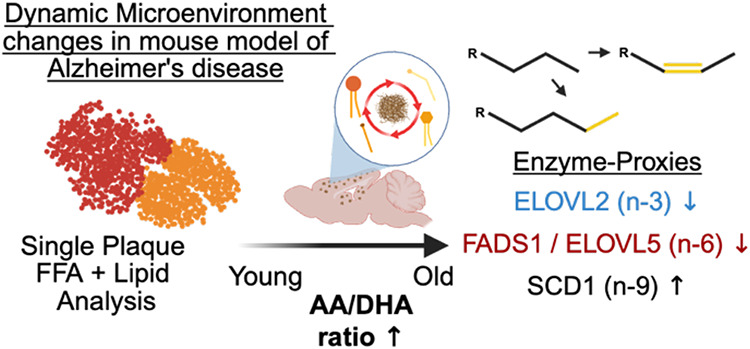

## Introduction

Free fatty acids (FFAs) are bioactive lipid species central to membrane remodeling, lipid droplet biogenesis, mitochondrial β-oxidation, and inflammatory signaling,^1–3^ positioning them as mechanistic participants in many cellular processes dysregulated in Alzheimer’s disease (AD). Altered FFA profiles correlate with cognitive decline and neuropathological burden in AD brain tissue and peripheral fluids.^4,5^ Lipid droplet accumulation in astrocytes and microglia links FFA metabolism directly to APOE-dependent Aβ clearance failure,^6–8^ while saturated fatty acid excess exacerbates Aβ accumulation, oxidative stress, and tau hyperphosphorylation in preclinical models.^9–11^

Despite this, spatially resolved mapping of FFAs in brain tissue has not been achieved. Conventional FFA analysis relies on GC–MS or LC–MS, bulk techniques that result in the loss of spatial information. Standard MALDI matrices, which have defined spatial lipidomics across cancer, neurodegeneration, and organ biology,^12–14^ do not support FFA ionization, and alternative strategies such as DESI (and nano-DESI) yield few FFA species with pronounced delocalization.^15^ The bioactive role of fatty acids at Aβ plaques has been anticipated by studies reporting plaque-associated changes in fatty acid composition within complex lipids—including enrichment of PUFA-containing phosphoinositols (PIs) and phosphatidic acids (PAs),^16–21^ and depletion of unsaturated acyl chains within phosphatidylcholines (PCs).^22^ Yet despite their divergent findings, these observations share a fundamental limitation: they characterize fatty acids only as esterified constituents of intact lipids, not as free, bioactive species capable of independent signaling. Released FFAs generate bioactive derivatives, such as prostaglandins, leukotrienes, and resolvins, via COX/LOX pathways.^23,24^ These molecules mediate inflammatory and pro-resolving signaling that esterified lipids cannot recapitulate, making spatially resolved FFA mapping a distinct and unresolved challenge. This technical barrier has left the corresponding biological questions related to FFA unresolved.

Aβ plaques are not uniform lesions; they differ in peptide composition, structural organization, and microenvironmental context across brain regions and disease stages.^25–28^ Peptide-level analyses have shown that Aβ deposition undergoes a dynamic early phase before reaching compositional stability,^27,29^ yet whether the surrounding lipid landscape continues to evolve after peptide composition stabilizes is unknown. Individual plaques exert distinct effects on their local microenvironments,^17,18,20,26^ and because Aβ pathology propagates across brain regions with disease progression,^29–32^ these microenvironmental differences are likely both region-specific and stage-dependent. Whether FFA remodeling at plaques is spatially concentrated, stage-dependent, or encodes biological information orthogonal to Aβ peptide readouts cannot be addressed without spatially resolved FFA measurements.

To overcome this barrier, we developed a chemically tailored MALDI mass spectrometry imaging (MSI) workflow that enables robust FFA ionization while preserving the detection of complex lipids, such as phospholipids and glycosphingolipids. We applied this approach to the Arctic (E693G) and Swedish (KM670/671NL) double mutant tgAPPArcSwe mouse model, which develops early-onset, widespread Aβ plaque pathology across multiple brain regions.^33^ Critically, the underlying Aβ fibril structure in this model closely resembles that of sporadic AD,^34^ making it uniquely suited for studying disease-relevant plaque microenvironments. By combining spatially resolved FFA profiling with single-plaque annotation through the Single Plaque Microenvironment Analysis (SPMA) framework, product-to-precursor ratios computed from codetected FFA species were used as steady-state proxies for local elongase and desaturase activity, with age-dependent differences assessed by inferential modeling accounting for the pseudoreplication inherent in multiplaque sampling. The resulting enzymatic indices were subsequently evaluated by machine-learning-based classification to determine whether the inferred pathway-level alterations are sufficiently structured to encode plaque age and anatomical identity at single-plaque resolution.

This strategy revealed two previously inaccessible dimensions of FFA biology at Aβ plaque sites: overall spatial organization across the brain and progressive remodelling at the level of individual deposits. FFA distributions are not diffuse but instead form locally enriched, highly structured spatial compartments tied to regional cytoarchitecture, cell-type composition, and membrane metabolic state. Within the plaque microenvironment, dozens of FFA species are significantly remodelled relative to perilesional tissue, with reciprocal enrichment of short saturated and long-chain highly unsaturated species alongside depletion of long-chain monounsaturated and saturated FFAs, consistent with concurrent disruption of ELOVL-mediated elongation and FADS-mediated desaturation within the plaque niche. Strikingly, FFA composition accurately classifies plaque age, and this remodeling trajectory continues after Aβ peptide composition has stabilized, indicating an ongoing lipid-metabolic program that is invisible to conventional peptide-based readouts.^27,29^

## Results

### MALDI-MSI Maps Region-Specific Fatty Acid Distributions in the Brain

To characterize the spatial organization of free fatty acids in the brain and their relationship to Aβ pathology, we developed a MALDI-MSI workflow enabling simultaneous detection of FFA and other anionic lipids. Brain sections from young (9 months) and aged (18 months) tgAPPArcSwe mice were acquired, and four anatomically distinct regions, cortex (Ctx), hippocampus (Hipp), subiculum (Sub), and thalamus (Thal), were delineated by co-registration with fluorescence histological staining (Figure 1A). The workflow detected 29 structurally annotated FFA species spanning C14–C24, encompassing saturated, monounsaturated, and polyunsaturated species across the long-chain and very long-chain range. We first characterized how this FFA landscape varies across brain regions and cortical layers, before zooming into the Aβ plaque microenvironment to resolve age- and region-dependent remodelling at the level of individual deposits.

**Figure 1:**
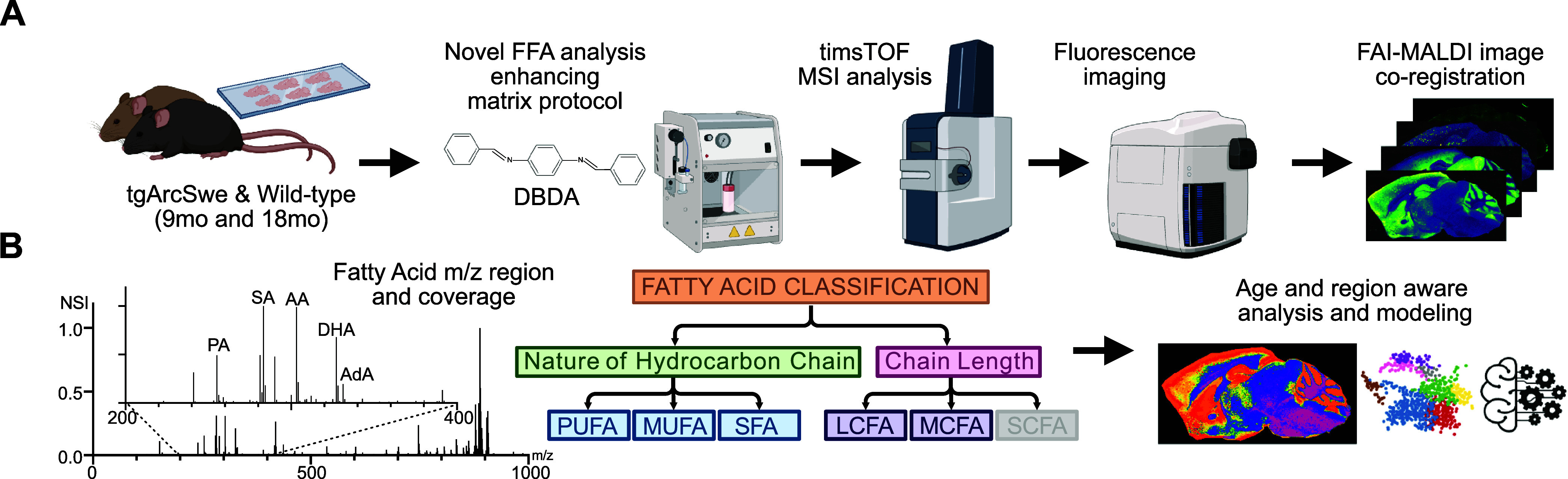
Experimental design and multimodal lipid imaging workflow. (A) Schematic overview of the WT and tgAPPArcSwe animal models (9 and 18 months) used to assess aging and AD pathology-associated lipid remodeling. Cryosections were analyzed by MALDI–MSI using the novel DBDA matrix protocol to enhance FFA detection, followed by timsTOF MSI acquisition, fluorescent amyloid imaging (FAI) for Aβ plaques, and spatial co-registration of MSI and fluorescence data. Bisecting k-means clustering of single-pixel spectra delineated major anatomical regions and revealed putative plaque-associated spectral features absent in WT animals. Automated feature detection and isotopologue correction were used to identify global metabolites and lipids, followed by manual curation of robustly detected and annotated species, including diverse phospho- and glycolipids as well as FFAs. (B) Representative negative-ion mode mass spectrum illustrating the *m*/*z* window used for FFA detection, with key species annotated, including palmitic acid (PA), stearic acid (SA), arachidonic acid (AA), docosahexaenoic acid (DHA), and adrenic acid (AdA). Detected FFAs were classified by the nature of their hydrocarbon chain (polyunsaturated, PUFA; monounsaturated, MUFA; and saturated, SFA) and by chain length (long-chain, LCFA; medium-chain, MCFA; and short-chain, SCFA). Multimodal data integration enabled plaque-centric quantification and machine-learning-based verification of regional and age-associated FFA alterations across brain regions and animal ages (created in https://BioRender.com).

Analysis of single-ion images showed clear regional organization in tgAPPArcSwe mouse brains. FFA species were enriched in specific anatomical structures, including the cortex, hippocampus, thalamus, and subiculum, and, in some cases, directly associated with Aβ plaques (Figure 2A). Shorter saturated LCFAs, including myristic acid (14:0), palmitic acid (16:0), and monounsaturated palmitoleic acid (16:1), showed relatively uniform distribution across gray matter regions (Figure 2B). In contrast, species with longer chains and varying degrees of unsaturation displayed pronounced regional specificity, with enrichment patterns differing within cortex (inner laminae and outer layers), within the hippocampus and subiculum (pyramidal and molecular layers of CA1–3, and granule/molecular layers of the dentate gyrus), and across the cerebellar molecular and granular layers, fibers tracts, and ventricular and capillary-rich areas (Figure 2C). Notably, similar patterns were also observed in wild-type animals (Figure S1), suggesting that the regional FFA landscape reflects normal brain architecture rather than Aβ pathology per se.

**Figure 2:**
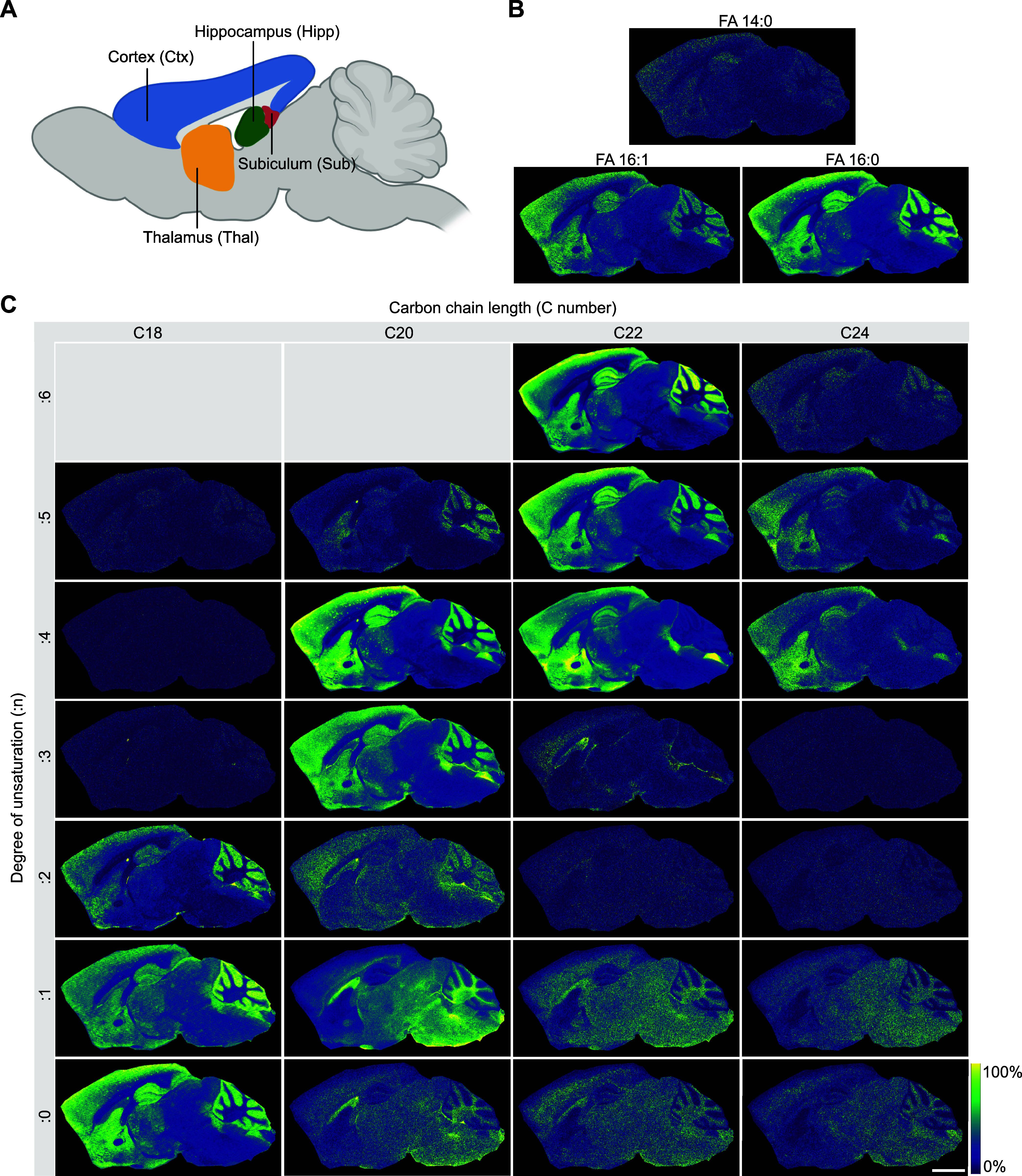
MALDI-MSI mapping of regional FFA distributions in mouse brain. (A) Schematic annotation of selected anatomical regions in the brain (Created in https://BioRender.com). (B) Representative single-ion images of shorter LCFA species, including myristic (14:0), palmitic (16:0), and palmitoleic (16:1) in old tgAPPArcSwe mice. (C) Spatial organization of FFAs grouped by carbon length (C18–C24) in rows, and degree of unsaturation in columns. Pronounced radial gradients were evident within cortex: saturated and monounsaturated C20–C24 species enriched in inner laminae, whereas polyunsaturated (4–6 double bonds) counterparts predominated in outer layers. Distinct laminar enrichment of highly unsaturated PUFAs was observed in the hippocampus and subiculum (pyramidal and molecular layers of CA1–3, and granule/molecular layers of the dentate gyrus). Cerebellar molecular and granular layers were enriched in polyunsaturated C20–C24 species, whereas fiber tracts contained higher monounsaturated signals. Ventricular and capillary-rich areas exhibited PUFA species with 2–3 double bonds. Scale bars: 2 mm; intensity scale: relative ion abundance (100% of maximum).

When organized by carbon chain length and degree of unsaturation, distinct spatial trends emerged. In the cortex, C20 LCFAs and C22–C24 VLCFAs showed pronounced radial gradients: saturated and monounsaturated species were enriched in inner cortical layers, while PUFA counterparts predominated in outer regions. For C18 species, this pattern reversed, with species carrying fewer double bonds concentrated in outer layers. These gradients suggest structured lipid compartmentalization tied to regional differences in membrane composition, cell-type distribution, and metabolic activity. In the hippocampus and subiculum, PUFAs with 4–6 double bonds localized to the pyramidal-associated layers of the Cornu Ammonis (CA1–3), while monounsaturated and less unsaturated C20–C24 FFAs were reduced in hippocampal areas relative to cortex, with the opposite pattern observed for C18 species. Beyond these regional patterns, a subset of FFA species showed clear colocalization with Aβ plaques, exhibiting region-specific differences in intensity and spatial extent. These included the saturated species myristic acid (14:0), palmitic acid (16:0), and stearic acid (18:0), as well as the polyunsaturated species arachidonic acid (AA, 20:4), adrenic acid (22:4), and docosahexaenoic acid (DHA, 22:6). Across other regions, saturated and monounsaturated C20–C24 species were enriched in cerebellar granular layer and white matter fiber tracts, while their polyunsaturated counterparts (≥3 double bonds) alongside less unsaturated C18 species (0–2 double bonds) showed preferential enrichment in the cerebellar molecular layer, mirroring the unsaturation-dependent laminar gradients observed in cortex.

### Region-Level Lipidomic Profiling Fails to Capture Plaque-Associated FFA Remodelling

To establish whether bulk regional analysis is sufficient to detect disease-associated lipid changes, we conducted a systematic quantitative comparison of regional FFA and lipid profiles between tgAPPArcSwe and wild-type animals. Global mean spectra were largely similar across regions, with spectral clustering confirming clear regional separation while age- and genotype-related differences remained minimal (Figure S2A,B). Bulk regional analyses, including single-pixel correlations and statistical comparison of individual species, revealed only modest trends (Figure S2C,D). Together, these analyses confirm that region-level comparisons lack the sensitivity to resolve spatially confined disease-relevant lipid remodeling. We therefore pursued a focused analysis of the local Aβ plaque microenvironment to resolve these spatially restricted alterations.

Co-registration of MALDI-MSI data with FAI enabled alignment of lipid ion distributions with individual Aβ deposits, and binary masks were used to annotate individual plaques and extract adjacent periplaque regions as local reference within the SPMA framework (Figures 3A and S3). Treating each annotated plaque as an individual analytical object, analogous to single-cell approaches in transcriptomics, this approach resolves plaque-to-plaque chemical variability independently of pixel-level noise and plaque size effects. This analysis identified nearly 100 successfully annotated lipid and FFA features (approximately 180 features total) significantly altered within plaques relative to their immediate surroundings, spanning sulfatides, cardiolipins, phospholipids, and FFAs, with both the direction and magnitude of alterations varying across brain regions and ages (Figure 3B and Table S1). Cortex in aged tgAPPArcSwe mice contained the largest number of uniquely enriched species, driven by distinct cardiolipins and FFAs, while the strongest shared intersections were observed between cortex and subiculum (Figure 3C). These findings demonstrate that single-plaque spatial resolution enables the detection of lipid remodeling associated with Aβ pathology that is obscured at the regional level and motivated the focused FFA enzymatic analysis of the plaque microenvironment described below.

**Figure 3:**
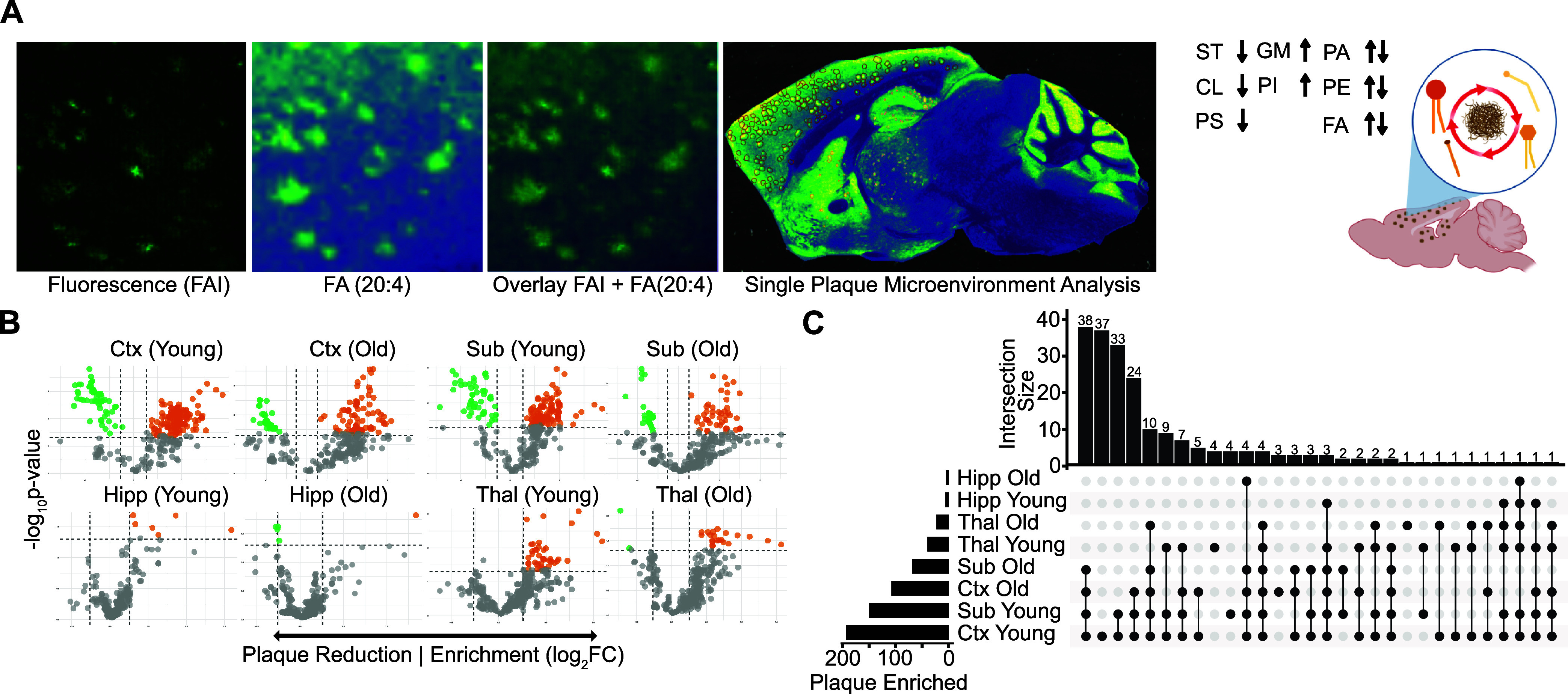
Single-plaque microenvironment analysis reveals region- and age-dependent lipid alterations at Aβ plaques. (A) Co-registration of FAI with single-ion MALDI-MSI maps enabled precise alignment of lipid distributions with individual Aβ plaques. Representative images show FAI signal, FA 20:4 ion map, and their overlay, illustrating near-perfect spatial colocalization. Whole-section SPMA was applied to extract plaque and periplaque ROIs across all annotated plaques. The schematic summarizes directional alterations of major lipid classes at the plaque microenvironment, including decreases in sulfatides (ST), chlorolipids (CL), and phosphatidylserines (PS), increases in gangliosides (GM) and phosphatidylinositols (PI), and bidirectional changes in phosphatidylethanolamines (PE), phosphatidic acids (PA), and free fatty acids (FFA) (created in https://BioRender.com). (B) Volcano plots depicting lipid and FFA features significantly altered between plaque and periplaque ROIs across all eight region-age comparisons (orange: plaque-enriched; green: plaque-reduced). Cortex and subiculum display the greatest number of significantly altered species, with consistent enrichment patterns between ages within each region. (C) UpSet plot of plaque-enriched lipid species across all region-age groups. Cortex in young mice harbors the largest set, with the strongest intersections between cortex and subiculum, reflecting shared plaque-associated remodeling. Analysis across six animals, approximately 300 plaques/animal (old) and 100 plaques/animal (young). Cutoffs: *p* < 0.05, |log_2_FC| > 1.2.

### Plaque-Associated FFA Remodeling Shows Subcortical Suppression of n-6 Elongation with Compensatory n-9 Desaturation

To characterize the compositional landscape of plaque-associated FFA remodeling, we examined the overall structure of significantly altered FFA species across regions and ages through hierarchical clustering (Figure 4A). This revealed two compositionally distinct groups. The left cluster comprised VLC-MUFA and VLC-SFA species, which were strongly enriched in the thalamus, most markedly in old mice, and markedly depleted in the hippocampus and subiculum of young mice. The right cluster encompassed the full PUFA spectrum, including shorter-chain species, separating into two subclusters: A highly unsaturated PUFA subcluster comprising long- and very long-chain polyunsaturated species; and a broader short-chain subcluster containing C14–C18 saturated and monounsaturated species, together with their partially elongated, less unsaturated C20–C24 intermediates. UMAP dimensionality reduction of the full single-plaque FFA matrix revealed partial separation by age alongside a pronounced regional structure, with thalamic and cortical plaques occupying largely nonoverlapping regions of the embedding, while hippocampal and subicular plaques showed intermediate positioning consistent with their shared subcortical enzymatic profile (Figure 4B). Together, the coherence of these regional and age-dependent patterns across both hierarchical clustering and UMAP structure suggested coordinated enzymatic regulation of plaque-associated FFA composition rather than independent species-level variation. To infer changes in local fatty acid processing, we therefore quantified product-to-precursor ratios as steady-state proxies for elongase and desaturase activity across individually annotated Aβ plaques. These ratios are grounded in two core biochemical reactions: desaturation, catalyzed by FADS and SCD family enzymes, which introduce a double bond via hydrogen abstraction (–H_2_); and chain elongation, catalyzed by ELOVL family enzymes, which extends the acyl chain by a two-carbon unit (+C_2_H_4_) (Figure 4C). Individual FFA species reflect the net outcome of multiple competing enzymatic and nonenzymatic processes and are not uniquely attributable to specific enzymatic steps; product-to-precursor ratios instead provide a mechanistically constrained readout of pathway-level flux, assessed here at the level of individual plaques while accounting for the multianimal pseudoreplication structure of the data set.

**Figure 4:**
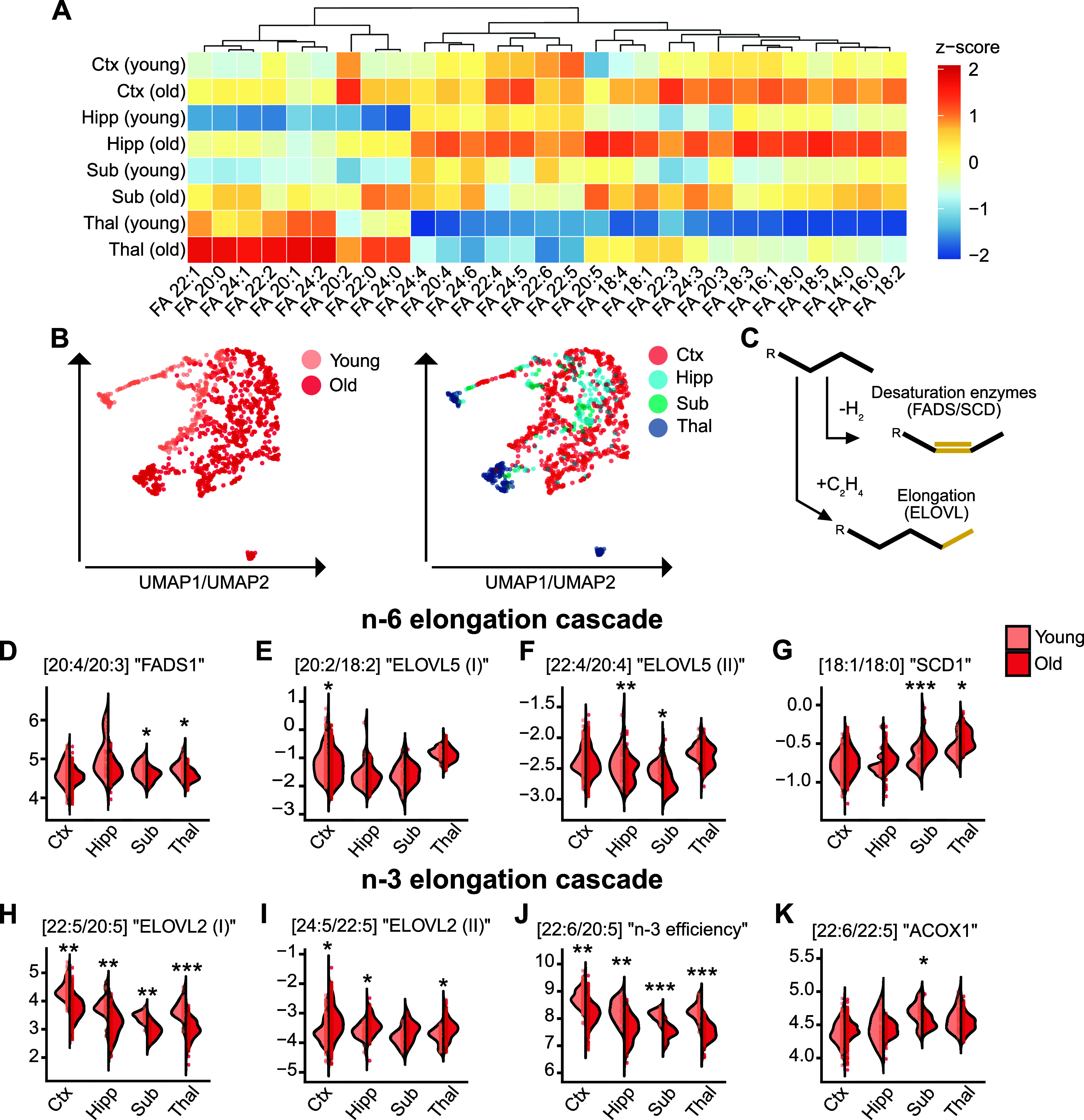
Plaque-associated FFA profiles reveal region- and age-dependent remodeling of desaturation and elongation pathways. (A) Hierarchical heatmap of all detected FFA species across the eight region-age groups, illustrating distinct FFA compositional signatures between cortex/subiculum and hippocampus/thalamus, and age-dependent shifts within each region. (B) UMAP embeddings of single-plaque FFA profiles colored by age and brain region, demonstrating that both age and region contribute to the structuring of plaque FFA space. (C) Simplified schematic of the enzymatic reactions underlying FFA remodeling, illustrating desaturation (loss of H_2_ FADS/SCD enzymes) and two-carbon chain elongation (addition of C_2_H_4_ ELOVL enzymes)­(Created in https://BioRender.com). (D–G) Enzymatic index ratios for the n-6 elongation cascade are shown as split-half violin plots (young/old) across all four regions. (D) FADS1 proxy [20:4/20:3] and (E) ELOVL5-mediated elongation steps proxies [20:2/18:2] and (F) [22:4/20:4] are predominantly suppressed in subiculum with age. (G) SCD1 proxy [18:1/18:0] shows compensatory upregulation selectively in the subiculum and thalamus. (H–K) Enzymatic index ratios for the n-3 elongation cascade. ELOVL2 proxies at both elongation steps (H) [22:5/20:5] and (I) [24:5/22:5] are broadly suppressed across all regions with age. The composite n-3 efficiency index (J) [22:6/20:5] confirms consistent suppression of DHA biosynthetic efficiency across regions. (K) ACOX1 proxy [22:6/22:5] shows a significant age effect restricted to the subiculum. Analysis across six animals, approximately 300 plaques/animal (old) and 100 plaques/animal (young). All comparisons performed at the animal level; *p < 0.05, **p < 0.01, ***p < 0.001.

Within the n-6 elongation cascade, the DGLA-to-AA ratio (20:4/20:3, proxy for FADS1-mediated Δ5-desaturation) was significantly reduced in subcortical regions, including subiculum and thalamus, with a strong trend in hippocampus (Figure 4D). Two independent ELOVL5 substrate-product ratios were concurrently reduced: the upstream EDA/LA ratio (20:2/18:2; designated ELOVL5 I as a C18 n-6 elongation proxy, noting that this branch is upstream of the canonical GLA-to-DGLA step) was reduced in cortex, while the adrenic/AA ratio (22:4/20:4, ELOVL5 II) was reduced in hippocampus and subiculum with a consistent trend in thalamus (Figure 4E,F). Cortical plaques showed no significant reduction in either FADS1 or ELOVL5 II, establishing a subcortical-to-cortical gradient in n-6 cascade suppression. Substrate-product correlation analysis further supported this interpretation: while EDA/LA coupling was strong across hippocampus, subiculum, and thalamus, it was essentially absent in cortex for both young and old mice (Figure S4A), indicating that LA-to-EDA conversion in cortex is not substrate-driven, consistent with enzymatic suppression of ELOVL5 I activity rather than reduced substrate availability. One alternative explanation for the observed ratio changes is PLA2-mediated release of AA from membrane phospholipids, which would be expected to elevate the free AA pool independently of elongase activity. AA-containing PI species showed no age-dependent changes in any region (Figure S4B,C), and free AA (FA 20:4) showed no consistent age-dependent elevation across regions and was significantly increased rather than depleted in hippocampus at old plaque sites (Figure S4D), together arguing against net phospholipase-driven AA mobilization as a confounding mechanism. Concurrently, the oleic/stearic ratio (18:1/18:0, SCD1) was elevated in subiculum and thalamus (Figure 4G), suggesting preferential Δ9-desaturation of the C18 substrate as a homeostatic response to long-chain PUFA depletion. The palmitoleic/palmitic ratio (16:1/16:0) was unchanged across all regions (Figure S4E), confirming substrate specificity and arguing against generalized SCD1 upregulation. Substrate-product correlation analysis corroborated this finding, showing systematic age-dependent separation in subiculum and thalamus for the stearic/oleic pair (Figure S4F).

### Dual Index-Based Signature Indicates Coordinated Suppression of n-3 DHA Biosynthetic Capacity Uniformly across Aβ Plaque Sites

In contrast to the regionally restricted pattern of n-6 suppression, the n-3 elongation cascade showed uniform impairment across all four brain regions of a fundamentally different character. Within the n-3 elongation cascade, ELOVL2-mediated elongation was broadly suppressed at aged plaque sites across both sequential substrate-product steps. The EPA-to-DPA ratio (22:5/20:5, ELOVL2 I) was significantly reduced in all four regions, with the strongest effects in the thalamus and cortex (Figure 4H). The downstream DPA-to-tetracosapentaenoic acid ratio (24:5/22:5, ELOVL2 II) was similarly reduced in the cortex, hippocampus, and thalamus (Figure 4I), confirming that suppression of ELOVL2 activity extends across successive elongation steps rather than being confined to a single substrate pair. A composite n-3 efficiency index (22:6/20:5) capturing the net conversion of EPA toward DHA was significantly reduced across all four regions (Figure 4J), providing an integrated measure of the pathway-level flux suppression. In contrast, the DHA/DPA ratio (22:6/22:5; designated ACOX1 as a Sprecher retroconversion index, reflecting the multistep peroxisomal completion of DHA synthesis rather than ACOX1 activity in isolation) was reduced only in subiculum (Figure 4K), suggesting that peroxisomal retroconversion contributes regionally to the observed DHA depletion but is not the dominant mechanism systemically. DHA-containing PI species were significantly reduced at old plaque sites specifically in the cortex (Figure S4G,H), while free DHA (FA 22:6) showed no age-dependent changes in any region (Figure S4I). This dissociation, reduced DHA-containing PI without a corresponding change in free DHA, argues against PLA2-mediated phospholipid hydrolysis and instead points toward cortex-specific remodeling of DHA-containing PI independent of free DHA availability. Together, these findings support coordinated enzymatic suppression of both n-6 and n-3 elongase and desaturase activity at aged plaque sites, with a subcortical predominance, indicating region-specific vulnerability of this processing machinery.

### Coordinated Shift in Plaque-Associated FFA Compositional Indices and Machine Learning Validation of the Enzymatic Signature

Having characterized the individual enzymatic steps underlying n-6 and n-3 remodeling at Aβ plaque sites, we next asked how these changes manifest in integrated compositional indices and whether the combined enzymatic signature is sufficiently structured to classify plaque identity at single-plaque resolution. The net shift in PUFA balance at aged plaque sites was visualized by mapping the AA/DHA ratio across tissue sections from representative young and old animals (Figure 5A). A systematic elevation of the AA/DHA ratio was apparent at plaque-associated regions in old relative to young tissue. This was consistent with a disproportionate collapse of n-3 relative to n-6 elongation at sites of mature Aβ deposition, reflecting the greater magnitude and regional breadth of ELOVL2 suppression described above rather than active n-6 upregulation. Quantification across individually annotated plaques confirmed this pattern across the cortex, subiculum, and thalamus (Figure 5B). The elongation capacity index (EC, ΣC20–24/ΣC14–18) was reduced across all four regions (Figure 5D), indicating a uniform shift toward shorter-chain FFA profiles with age. The unsaturation index (UI, ΣPUFA/ΣSFA) was reduced in subiculum and thalamus, with consistent trends in cortex and hippocampus (Figure 5C), reflecting reduced net desaturase output concentrated in subcortical regions.

**Figure 5:**
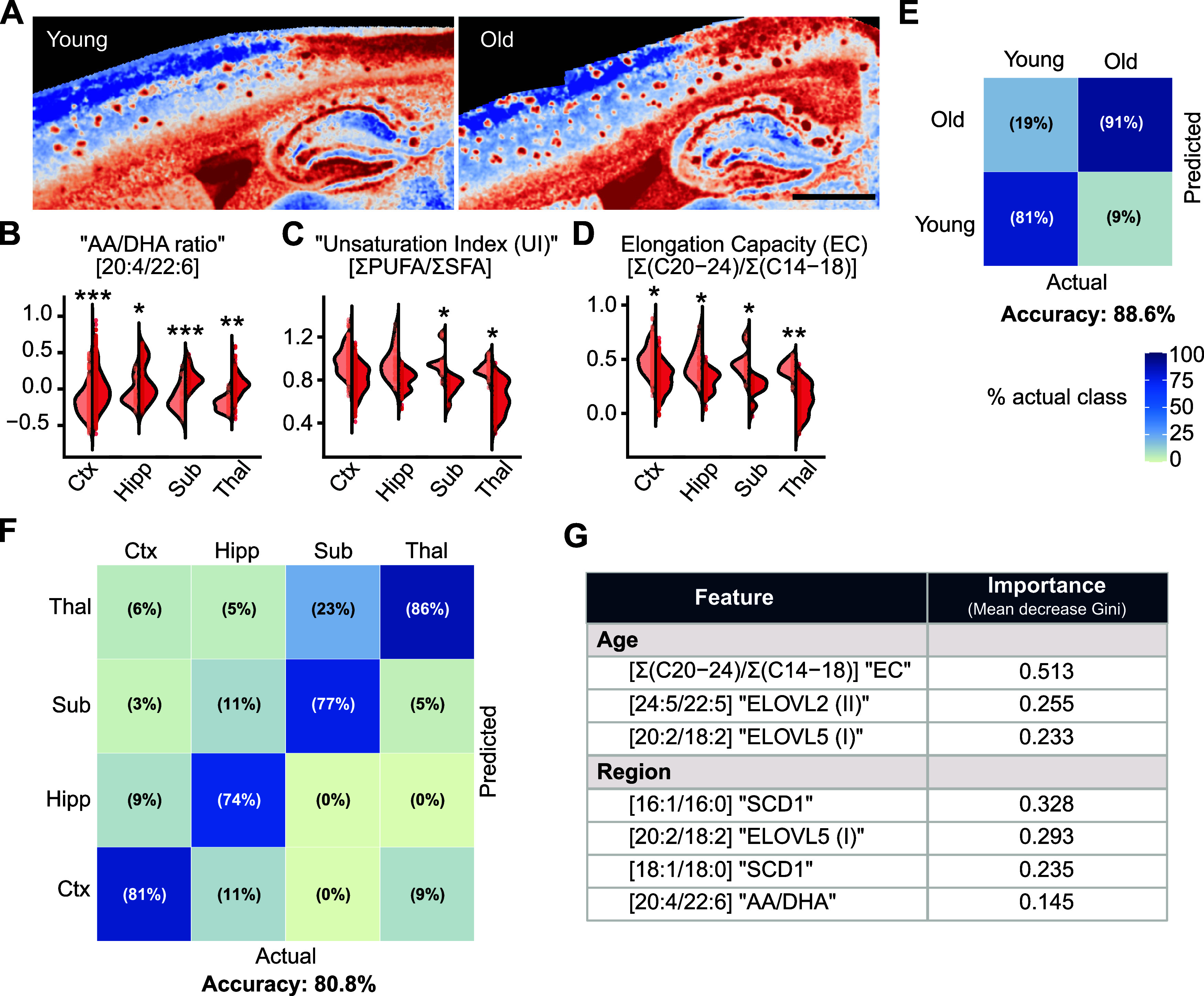
Composite FFA indices capture age- and region-dependent enzymatic remodeling at Aβ plaques and support machine-learning-based classification. (A) Spatial maps of the AA/DHA ratio [20:4/22:6] in young and old tgAPPArcSwe brain sections, illustrating the age-dependent shift toward AA dominance across plaque-bearing regions. Scale bar: 1 mm. (B–D) Split-half violin plots across all four regions for three composite FFA indices: (B) AA/DHA ratio [20:4/22:6], (C) Unsaturation proxy Index (UI; ΣPUFA/ΣSFA), and (D) Elongation Capacity proxy (EC; Σ­(C20–24)/Σ­(C14–18)). All three indices show significant age-dependent alterations, with the AA/DHA ratio elevated across all regions and EC predominantly reduced in the subiculum and thalamus. (E) Confusion matrix for a minimal 3-feature machine learning classifier of plaque age, achieving 88.6% accuracy. The top age-discriminating features were EC proxy [Σ­(C20–24)/Σ­(C14–18)], ELOVL2 (II) proxy [24:5/22:5], and ELOVL5 (I) proxy [20:2/18:2]. (F) Confusion matrix for a minimal 4-feature machine learning classifier of plaque brain region, achieving 80.8% accuracy, with the highest classification performance for cortex and subiculum. (G) Mean Gini decrease feature importance for the minimal-age and region classifiers. Elongation capacity proxy dominates age classification, while SCD1 proxy indices and ELOVL5 proxy drive region discrimination. Analysis across six animals, approximately 300 plaques/animal (old) and 100 plaques/animal (young). All comparisons at animal level; *p < 0.05, **p < 0.01, ***p < 0.001.

To evaluate whether the enzymatic indices encode sufficient information to classify plaque age and brain region at a single-plaque resolution, we applied machine-learning-based classification to the complete set of enzymatic ratio indices. Models were trained by using sample-size balancing to account for unequal plaque counts across age groups and regions. The full-panel age model achieved 93.7% accuracy, with robust separation of young (93%) and old (98%) plaques (Figure S5A). The full-panel region model achieved 80.1% accuracy, with thalamus (93%) and subiculum (85%) showing the highest per-region sensitivity, consistent with their strong enzymatic alterations in LME analysis (Figure S5B). We next asked whether this signal was concentrated in a few dominant indices or was distributed across the enzymatic cascade. This distinction informs whether a minimally interpretable panel could serve as a proxy for the full enzymatic signature. An exhaustive combination search across all subsets of up to four indices showed that no minimal combination matched full-panel age performance and that region classification was recoverable with four features, indicating a distributed rather than focally concentrated signal for age and a more compact but cross-pathway structure for region. This distributed structure likely reflects the biochemical interdependence of fatty acid elongation and desaturation pathways, where coordinated enzymatic regulation creates inherent covariance across ratio indices. A cross-pathway age panel comprising EC, ELOVL2 (II), and ELOVL5 (I) achieved 88.6% accuracy (Figure 5E), and a cross-pathway region panel comprising SCD1 (16:1/16:0), ELOVL5 (I), SCD1 (18:1/18:0), and AA/DHA achieved 80.8% (Figure 5F,G). Critically, both cross-pathway panels outperformed the importance-ranked feature sets of equivalent size (age: 83%, Figure S5C; region: 72.7%, Figure S5D), with the region panel gaining performance specifically by substituting ELOVL2 (I) for the cross-cascade AA/DHA ratio, which captures the relative n-6/n-3 balance rather than a single elongation step. Together, these findings show that plaque-associated FFA remodeling is defined by age-dependent suppression of long-chain PUFA elongation across regions with additional desaturation changes concentrated in subcortical plaque microenvironments. The structured, region-specific nature of these shifts argues against nonspecific lipid release and instead supports coordinated enzymatic remodeling across multiple pathways.

## Discussion

The present study establishes spatially resolved FFA profiling at the single-Aβ-plaque level as a technically and conceptually distinct analytical dimension in AD research. By combining chemically optimized MALDI-MSI with single-plaque annotation, it resolves enzymatic lipid remodeling within the Aβ plaque microenvironment that is inaccessible to conventional bulk approaches. The DBDA matrix enables robust negative-mode FFA ionization and complex lipid detection in a single acquisition, a combination not achievable with standard MALDI matrices. Protocol-level innovations in matrix chemistry, deposition, and acquisition parameters enhance FFA ion yield and stability across C14–C24 species at single-Aβ-plaque spatial scales while preserving coverage of phospholipids, sphingolipids, and glycolipids. The SPMA framework extends this by treating each plaque as an independent analytical unit, analogous to single-cell approaches in transcriptomics, enabling statistical decomposition of plaque-to-plaque variability that is lost in pixel- or region-level analyses. Together, these advances open a previously inaccessible analytical window on the plaque microenvironment.

The detected FFA species spanning C14–C24 were not uniformly distributed but formed structured laminar and region-specific compartments across the brain. Within the cortex, saturated and monounsaturated C20–C24 species were concentrated in the inner cortical layers, while PUFAs predominated in the outer laminae. This pattern mirrors known gradients in cortical cytoarchitecture: deeper, myelin-rich layers favor structurally rigid, saturated lipids that support axonal insulation,^35,36^ whereas the more neuronally dense superficial layers contain higher proportions of PUFAs that contribute to membrane fluidity, synaptic vesicle dynamics, and lipid-mediated signaling.^37^ In the hippocampus and subiculum, distinct PUFA enrichment was observed within the pyramidal-associated layers of CA1–3 and the molecular and granule layers of the dentate gyrus, consistent with their high synaptic density and signaling activity.^37^ These spatial patterns indicate structured lipid compartmentalization throughout the brain, reflecting underlying differences in membrane composition and metabolic activity, and are general to both tgAPPArcSwe and wild-type animals.

Despite these pronounced spatial gradients, the overall FFA landscape remained remarkably consistent across age and genotype when averaged at the regional level. This stability suggests that major lipid classes and regional FFA pools are preserved once brain development is complete, implying that disease-related lipid dysregulation manifests at a more localized scale, within the immediate Aβ microenvironment, rather than as broad shifts across regions or ages. Single-plaque microenvironment analysis confirmed this, identifying approximately 30 FFA and diverse lipid features that were significantly altered within plaques relative to their immediate surroundings, with both the direction and magnitude of these alterations varying across brain regions and ages. These results demonstrate that single-plaque spatial resolution enables the detection of lipid remodeling associated with Aβ pathology that is not detectable at the regional level.

Previous peptide-centric studies, including our own, have shown that Aβ deposition undergoes an early dynamic phase,^29^ followed by compositional stabilization,^27^ after which plaque numbers increase without major changes in peptide makeup. In contrast, our FFA-focused analysis reveals that lipid metabolism remains active and adaptable, continuing to remodel the microenvironment as seen observed by comparing the Aβ plaques in young and old tgAPPArcSwe animals. This dissociation between static peptide cores and dynamic microenvironment reflecting lipid profiles highlights how FFA metabolism tracks the ongoing biological activity of plaques, capturing cellular and inflammatory processes invisible to Aβ peptide readouts.

To characterize the enzymatic basis of this remodeling, we interpreted product-to-precursor ratios as steady-state proxies for elongase and desaturase flux, mechanistically constrained readouts of pathway-level activity rather than direct enzyme measurements, assessed across individually annotated plaques. The concurrent reduction in FADS1 [20:4/20:3] at subcortical plaques and ELOVL5 I [20:2/18:2] in cortex, alongside ELOVL5 II [22:4/20:4] in hippocampus and subiculum, points toward suppression of endogenous AA biosynthesis from dietary precursors rather than phospholipase-mediated release. cPLA2 is upregulated in AD brain and activated at Aβ plaques,^38,39^ and if the primary driver were PLA2-mediated AA mobilization, one would expect depletion of AA-containing phospholipid species and a broad elevation of free AA. Neither was observed: AA-containing PI species showed no age-dependent changes in any region, and free AA was not consistently elevated across regions. This argues against net phospholipase activity as a confound and instead supports a model of primary elongase and desaturase suppression, in which ratio changes reflect reduced biosynthetic throughput rather than altered phospholipid hydrolysis, consistent with evidence for dysregulation of fatty acid desaturase and elongase pathways in AD brain.^40–42^ The cortical sparing of FADS1 and ELOVL5 II changes, and the absence of substrate-product coupling in cortex, further indicate that these signatures are not a generic response to Aβ deposition but reflect region-specific features of the lipid metabolic environment, potentially differences in cell-type composition, baseline lipid metabolism, or local neuroinflammatory state, consistent with the known pattern of regional metabolic vulnerability in human AD.^43–45^

The elevation of the SCD1 index (oleic/stearic, 18:1/18:0) in subiculum and thalamus, directionally opposite to FADS1 and ELOVL5, points to activation of the unfolded protein response as a concurrent and mechanistically distinct component of plaque-associated lipid remodeling in subcortical regions. SCD1 is a direct transcriptional target of the UPR master regulator XBP1s^46,47^ and is upregulated under ER stress conditions to increase the proportion of monounsaturated fatty acids in membrane phospholipids, reducing membrane rigidity and alleviating ER stress-induced apoptotic signaling. ER stress is well-documented in neurons and astrocytes within the Aβ plaque microenvironment, and UPR activation has been reported in both human AD tissue and APP transgenic mouse models at ages corresponding to established plaque pathology.^48,49^ The opposing directions of SCD1 elevation and FADS1/ELOVL5 suppression in the same subcortical regions suggest that plaque-associated ER stress and neuroinflammatory signaling impose distinct metabolic demands on fatty acid remodelling, with SCD1 upregulation representing a homeostatic compensatory response to PUFA depletion rather than an independent pathological process. The substrate specificity of the SCD1 response, confined to the C18 substrate pair and absent for C16, further supports a targeted rather than generalized upregulation of Δ9-desaturation activity.

In contrast to the regionally restricted n-6 pattern, ELOVL2-mediated elongation showed uniform impairment across all four brain regions, establishing a fundamentally distinct and spatially broader axis of DHA biosynthetic failure.^50^ The magnitude and region-invariant nature of ELOVL2 suppression indicate a broad reduction in the elongation of EPA to DPA and, ultimately, to DHA.^51^ This pathway underpins the biosynthesis of pro-resolving lipid mediators, including resolvins and protectins.^52^ Reduced DHA availability and impaired lipid mediator resolution have been repeatedly documented in AD brain and proposed as contributors to chronic neuroinflammation in AD.^53–55^ The present data extend these observations to the single-plaque level, establishing that DHA biosynthetic suppression is a consistent feature of the aged plaque microenvironment across anatomical regions rather than a region- or stage-specific finding. Notably, ELOVL2 is among the most reproducible epigenetic aging clock genes, with promoter methylation-driven silencing of ELOVL2 expression reported across tissues with advancing age,^56,57^ suggesting that the anatomically uniform DHA biosynthetic deficit observed here may reflect a broader aging-associated transcriptional program rather than a plaque-specific response. The dissociation between reduced DHA-containing PI species specifically in the cortex and unchanged free DHA across all regions argues against PLA2-mediated phospholipid hydrolysis as the driver of cortical PI-DHA depletion and instead points toward cortex-specific remodeling of DHA-containing PI through nonhydrolytic mechanisms, potentially including altered PI biosynthesis, lysophospholipid reacylation, or lipophagy, that operate independently of free DHA availability. This cortex-specific PI-DHA finding represents a spatially distinct layer of lipid remodeling that is not captured by free FFA measurements alone and represents a candidate mechanism for future targeted investigations.

Machine-learning-based classification of individual plaques using the complete enzymatic ratio panel provides orthogonal support for the enzymatic signature identified by LME analysis and reveals its distributed nature. The full-panel age and region models both achieved robust accuracy, with the thalamus and subiculum showing the highest per-region sensitivity, consistent with their strong enzymatic alterations in LME analysis. No minimal feature subset matched full-panel performance, confirming a distributed rather than a focal signal. In both classifiers, cross-pathway panels outperformed importance-ranked feature sets of equivalent size, establishing that pathway diversity rather than individual feature strength is the key determinant of the classification performance. This distributed structure reflects the inherent biochemical covariance of the elongation and desaturation cascades, where shared substrates, competing enzymatic machinery, and coordinated transcriptional regulation link the ratio indices across pathways. The superior performance of cross-pathway panels, spanning global chain-length output (EC), terminal n-3 elongation flux (ELOVL2 II), and n-6 elongation entry (ELOVL5 I) for age and SCD1 activity, ELOVL5 elongation, and cross-cascade n-6/n-3 balance for region, demonstrates that the plaque-associated FFA signature is encoded across multiple biochemical axes simultaneously.

The present findings reveal a spatially and temporally structured program of enzymatic lipid remodeling within the Aβ plaque microenvironment that is invisible at the regional level of analysis. Two interlinked axes emerge. First, a regionally graded axis of n-6 elongase and desaturase suppression, with ELOVL5 I restricted to cortex and FADS1/ELOVL5 II concentrated in subcortical regions, accompanied by compensatory SCD1-mediated MUFA synthesis, reflects the neuroinflammatory and ER stress state of plaque-bearing subcortical tissue. Second, a region-spanning axis of n-3 elongation failure uniformly suppresses DHA biosynthetic flux across all regions and intensifies with Aβ maturation. These findings are consistent with, and provide enzymatic-level mechanistic grounding for, prior reports of altered PUFA metabolism, impaired lipid mediator resolution, and ceramide elevation in human AD brain, while extending them to the single-plaque spatial scale. This dissociation between the regional scope of n-6 and n-3 suppression has important implications for understanding the lipid metabolic basis of regional vulnerability in AD. The subcortical concentrations of n-6 elongase and desaturase change, most pronounced in the subiculum and thalamus, align with the known distribution of early and severe plaque pathology and neuroinflammatory activation in the tgAPPArcSwe model at 18 months, as well as with the regional pattern of metabolic vulnerability in human AD. In contrast, the region-invariant suppression of ELOVL2 suggests that DHA biosynthetic failure is a systemic feature of the plaque microenvironment that is not modulated by regional context, potentially reflecting a shared upstream regulatory mechanism, such as age-associated ELOVL2 promoter methylation or substrate competition with the n-6 pathway, that operates across all plaque-bearing regions irrespective of local inflammatory state.

Beyond AD, the workflow can be readily adapted to other neurodegenerative or inflammatory conditions characterized by localized metabolic reprogramming. Within AD, our findings position FFAs as active reporters of enzymatic activity within the plaque niche, linking Aβ deposition, glial metabolism and lipid turnover through a spatially structured and age-progressive remodelling program. Together, these data establish spatial FFA profiling using enhanced MALDI-MSI as a next-generation tool for deciphering lipid-driven mechanisms of Aβ pathology and for guiding therapeutic strategies that target the metabolic and immune dimensions of AD.

## Methods

### Chemicals and Reagents

All chemicals used in sample preparation were purchased from Sigma-Aldrich Sweden AB (Stockholm, Sweden) unless otherwise specified. N1,N4-Dibenzylidene benzene-1,4-diamine (DBDA) was synthesized and verified as previously described,^58,59^ and purity was determined to be greater than 95% (Figure S6). Deionized water was obtained from a Milli-Q purification system (Millipore Corp.).

### Animal Experiments

This study was conducted in 9- and 18-month-old transgenic mice (3 + 3) carrying both the Arctic and Swedish mutations in the human APP gene (tgAPPArcSwe), and age-matched wild-type (WT) C57bl6 mice. Mice were reared ad libitum at the animal facility at Uppsala University under a 12/12-hlight/dark cycle. All experiments were approved by the Uppsala Animal Ethics Committee (approval 5.8.18–20401/2020 and 5.8.18–16493/2024), and the study was conducted in accordance with the EU directive 2010/63/EU for animal experiments. For this study, no randomization, blinding, or sample-size calculations were performed.

### Tissue Sampling, Preservation, and Cryosectioning

The mice were anesthetized with isoflurane and euthanized by transcardial perfusion with saline before the isolation of brain tissue. The brains were dissected quickly with a 3 min post-mortem delay. Dissected tissue was split sagitally and snap frozen. Brain tissue samples were cryo-sectioned using a Leica CM3050 S cryostat (Wetzlar, Germany) at −23 °C with a thickness of 10 μm. Sections were thaw-mounted onto indium tin oxide (ITO) coated glass slides (Bruker Daltonics, Bremen, Germany) and stored at −80 °C until MALDI-MSI analysis.

### Sprayer-Based MALDI-MSI Sample Preparation

For matrix deposition, samples were thawed in a desiccator under reduced pressure for 30 min. Following method optimization, the N1,N4-dibenzylidenebenzene-1,4-diamine (DBDA) was applied to the tissue sections using the M3+ Sprayer (HTX Technologies, Chapel Hill). Briefly, 7 mg/mL DBDA in 70% acetone was sprayed onto tissue using the following instrumental parameters: nitrogen flow (10 psi), spray temperature (50 °C), 14 passes, offsets and rotations pattern (CC), spray velocity (1000 mm/min), and isocratic flow of 70 μL/min. During the experiments, the matrix was kept at 50 °C. The final matrix solvent composition, spray temperature, and number of passes were optimized on consecutive tissue sections.

### MALDI-MSI Data Acquisition

The MALDI-MSI experiments were conducted using a timsTOF fleX dual-source MALDI mass spectrometer (Bruker Daltonics, Bremen, Germany), in the negative-ion mode from 100–1600 Da, with a lateral resolution of 10 and 20 μm. The slides underwent height correction and focus adjustment. Transfer settings were 350 Vpp (funnel 1 RF), 350 Vpp (funnel 2 RF), and 350 Vpp (multipole RF). The focus pretime-of-flight (TOF) transfer time was set at 100 μs and prepulse storage at 5 μs. The quadrupole ion energy was 5 eV with a low mass of *m*/*z* 100. Collision cell energy was 10 eV with the collision RF set to 2500 Vpp. All of the spectra were recorded using a 10 kHz laser repetition rate.

MALDI-MS/MS was performed on tissue sections using collision-induced dissociation (CID) on a MALDI Fourier-transform ion cyclotron resonance (FTICR) (Solarix XR 7T-2ω, Bruker Daltonics, Bremen, Germany) mass spectrometer equipped with a Smart-beam II 2 kHz laser. The analysis was performed in quadrature phase detection (QPD) (2ω) mode; the time-of-flight (TOF) values were set at 0.5 ms; and the transfer optics frequency was 4 MHz. The isolation window was set to 1.0 *m*/*z*, and collision energies of 10–20 V, depending on the target parent ion. The mass resolution was calculated as ∼260,000 at *m*/*z* 350. The identified product ions were then compared to the product ion spectra of available standards, previously published studies, and/or interpreted fragmentation pathways of target molecules. External calibration was performed using red phosphorus (Red P) clusters for both the timsTOF and Solarix instruments.

### Fluorescent Amyloid Imaging (FAI)

Following MALDI analysis, tissues were rinsed in ice-cold absolute EtOH for 60s, then fixed in absolute EtOH for 5 min, rehydrated in 50% EtOH/MQ for 3 min, and rinsed for 5 min in PBS. As before, the tissue was then stained with luminescent conjugated oligothiophenes (LCO),^16,26^ the Amytracker 520 (Ebba Biotech, Solna, Sweden) at a 1 μg/mL dilution in PBS. Sections were then rinsed for 5 min in PBS and mounted with DAKO fluorescent mounting media (Agilent Technologies, Santa Clara, USA). Fluorescent imaging was performed on an automated widefield scanner (Axio Scan Z1, Zeiss, Jena, Germany). Large multichannel z-series tile scans were captured using a Plan-Apochromat 20×/0.8 DIC air objective and an ORCA monochrome camera (Hamamatsu Photonics, Hamamatsu, Japan). Excitation was provided by an LED module at 475 nm (illumination 450–488 nm), and emission was collected using a 504–546 nm filter set. Image processing and quantification were performed in QuPath v0.6.0 (open-source, GitHub).

### MALDI-MSI Data Annotation and Analysis

After the acquisition, data were imported and analyzed within the SCiLS Lab software (v.2025a Pro, Bruker Daltonics, Bremen, Germany). Initial preprocessing involving TopHat baseline subtraction and normalization to root-mean-square was performed. For the initial analysis, feature finding based on the T-ReX3 algorithm was employed to generate a feature table. Spatial segmentation using the Bisecting k-Means clustering algorithm in SCiLS was then conducted to facilitate brain region-specific segmentation and identification of putative Aβ plaque features. Pseudocoloring was applied by the software on a pixel-by-pixel basis, providing an initial visual representation of the chemical differences captured within distinct pseudoclusters.

For the bottom-up statistical analysis, R Studio was used. The MSI data were coregistered with the true pathological identification based on FAI through affinity transformation, and Aβ plaque regions of interest (ROIs) were identified using automated threshold-based plaque detection. An average of 300 plaques/animal (old) and 100 plaques/animal (young) were annotated across three individual animals of each age. At this point, a new feature list was created after refining to include only features enriched or reduced in Aβ. The putative feature annotation was done by matching accurate mass measurements (within a ± 3 ppm mass error tolerance) to the Lipid Maps database (https://www.lipidmaps.org/).^60–62^ Direct comparison was also made against previously reported lipid species specific to plaque pathology.^14–18,20,58,59^ This annotation was then refined in MetaboScape (v.2025b, Bruker Daltonics, Bremen, Germany) based on monoisotopic mass and ion mobility (collision cross-section, CCS) values, as previously described.^17^ The identity of features of interest was confirmed based on MSMS analysis as described above. The *m*/*z* values without annotations were retained in the analysis to maintain a mostly unbiased approach.

### Statistical Analysis

Statistical analyses were performed with R (v4.4.1) and GraphPad Prism 9. Linear mixed-effects models were used to test age and region effects on enzymatic indices, with animal as a random intercept to account for repeated measures across plaques. Batch correction was applied using the limma package (v3.60.3) prior to all downstream analyses. Pearson correlation was used for the substrate-product coupling analysis.

### Machine Learning-Based Classification

Random forest models were built using the randomForest package (v4.7–1.1) in R (v4.4.1), trained on single-plaque Limma-normalized data. Models were trained by using sample-size balancing to account for unequal plaque counts across age groups and regions. Each model was trained with 500 trees and default mtry settings. Model performance was evaluated on a stratified held-out test set (25%) with an out-of-bag error as an independent internal estimate. Full-panel models were trained on the complete set of enzymatic ratio indices. To assess the signal distribution, an exhaustive search across all combinations of up to four indices was performed to identify minimal feature panels. Cross-pathway panels were compared to importance-ranked feature sets of equivalent size. Confusion matrices and per-class accuracy were used to assess the classification performance for both age and region models.

## Supplementary Material



## Notes

Representative single-ion images of FFA in the old WT mouse brain (Figure S1); regional bulk lipid profiles across brain regions and ages in AD mice (Figure S2); co-registration of fluorescence Aβ imaging and MALDI-MSI (Figure S3); substrate-product correlations and phospholipid controls (Figure S4); ML classification of plaque identity using enzymatic proxy indices (Figure S5); NMR characterization of the DBDA matrix (Figure S6); and annotated features significantly altered in Aβ plaques (Table S1) (PDF)
