# Supplementary material for: Spatially Resolved Free Fatty Acid Profiling Reveals Region- and Age-Dependent Remodeling of Alzheimer’s Disease Plaques
Source: JACS Au. 2026 Jul 11;6(7):4153–65. doi: 10.1021/jacsau.6c00649 (PMC13417193; doi:10.1021/jacsau.6c00649)
Supplement: Supplementary file 1 [file au6c00649_si_001.pdf]

Supporting Information

Spatially Resolved Free Fatty Acid Profiling Reveals Region- and Age-Dependent Remodeling of Alzheimer's Disease Plaques

Sona Hakhverdyan[1], Sofie Hansson[1], Pascal Kadej[1], Sophia B. Orlovsky[2], Siarhei Hladkou[1], Birger Viirman[1], Anna Nilsson[3], Martin Lord[4], Stephanie M. Cologna[2,5], Justin T Mohr[2], Stina Syvänen[1], Per E. Andrén[3], Wojciech Michno[1,6,*]

1) Department of Public Health and Caring Sciences, Molecular Geriatrics, Uppsala University, SE-75237 Uppsala, Sweden

2) Department of Chemistry, University of Illinois Chicago, Chicago, Illinois 60607, United States

3) Department of Pharmaceutical Biosciences, Spatial Mass Spectrometry, Science for Life Laboratory, Uppsala University, SE-75123 Uppsala, Sweden

4) Department of Pharmacy, Science for Life Laboratory, Uppsala University, SE-75123 Uppsala, Sweden

5) Laboratory of Integrative Neuroscience, University of Illinois Chicago, Chicago, Illinois 60607, United States

6) Science for Life Laboratory, Uppsala University, Uppsala, SE-752 37, Sweden

* Correspondence: wojciech.michno@scilifelab.uu.se (W.M)

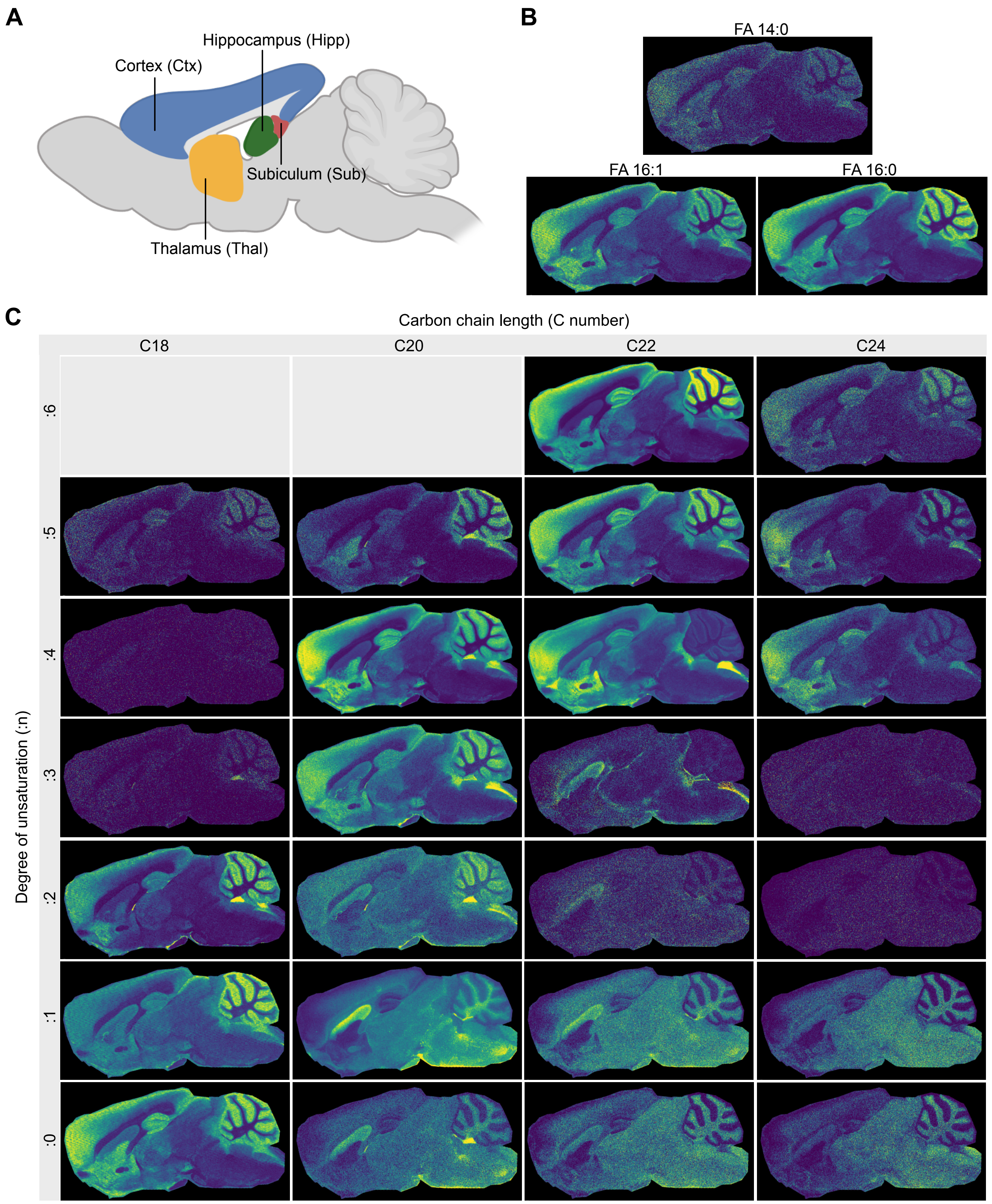

Supplementary Figure 1. Representative single-ion images of FFA in old WT mouse brain.

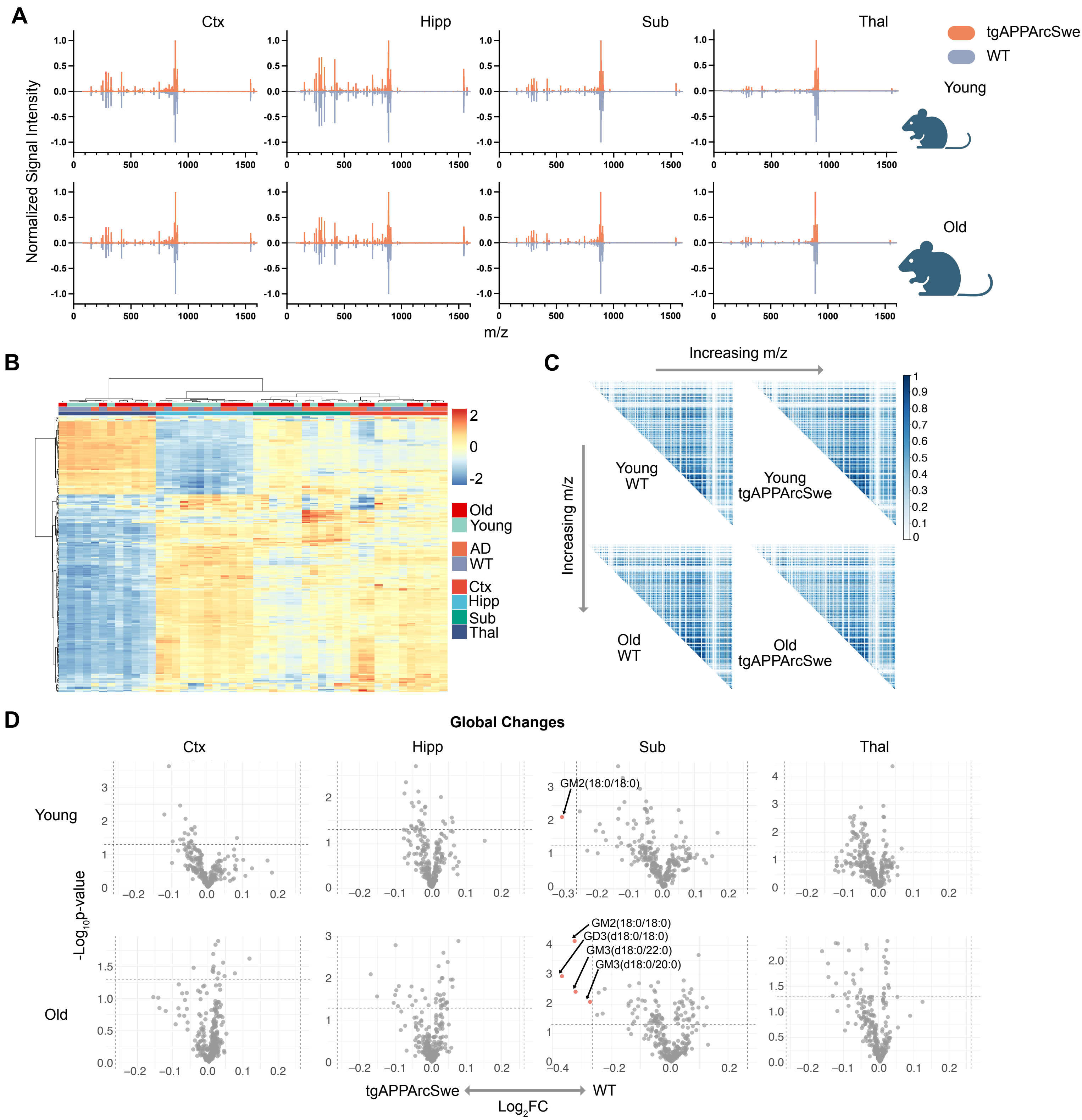

Supplementary Figure 2. Regional lipid diversity and absence of bulk AD pathology signal across brain regions and ages. (A) Mirror spectral profiles of normalized mean signal intensity for tgAPPArcSwe (orange) and WT (blue) across all four regions (Ctx, Hipp, Sub, Thal) at young (top) and old (bottom) ages, illustrating the dominant contribution of regional lipid composition over genotype across the full m/z range. (B) Hierarchical clustering heatmap of all detected lipid species across samples, demonstrating primary separation by brain region and secondary separation by age, with no consistent clustering by genotype. (C) Pairwise spectral correlation matrix across the four experimental groups (young WT, young tgAPPArcSwe, old WT, old tgAPPArcSwe), showing high intra-group coherence and region-driven inter-group structure. (D) Volcano plots of tgAPPArcSwe vs. WT comparisons across all four regions at young and old ages. No lipid species reach significance thresholds ($p < 0.05$, $|\log_2 FC| > 0.2$) in cortex, hippocampus, or thalamus at either age. Subiculum shows selective enrichment of ganglioside species (GM2, GD3, GM3) in old tgAPPArcSwe mice, consistent with focal plaque-associated signal detectable only in this region at bulk resolution.

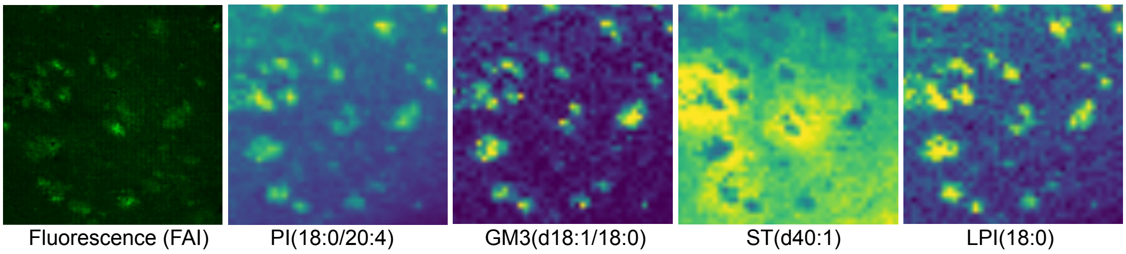

Supplementary Figure 3. Co-registration of fluorescence amyloid imaging and MALDI-MSI ion maps at single-plaque resolution. Fluorescence image (FAI, far left) marks individual Aβ plaques in a representative tgAPPArcSwe tissue section. Adjacent panels show MALDI-MSI ion images for four representative lipid species: PI(18:0/20:4), GM3(d18:1/18:0), ST(d40:1), and LPI(18:0); illustrating the spatial heterogeneity of plaque-associated lipid distributions and confirming pixel-level co-registration between the fluorescence and mass spectrometry modalities used for single-plaque annotation.

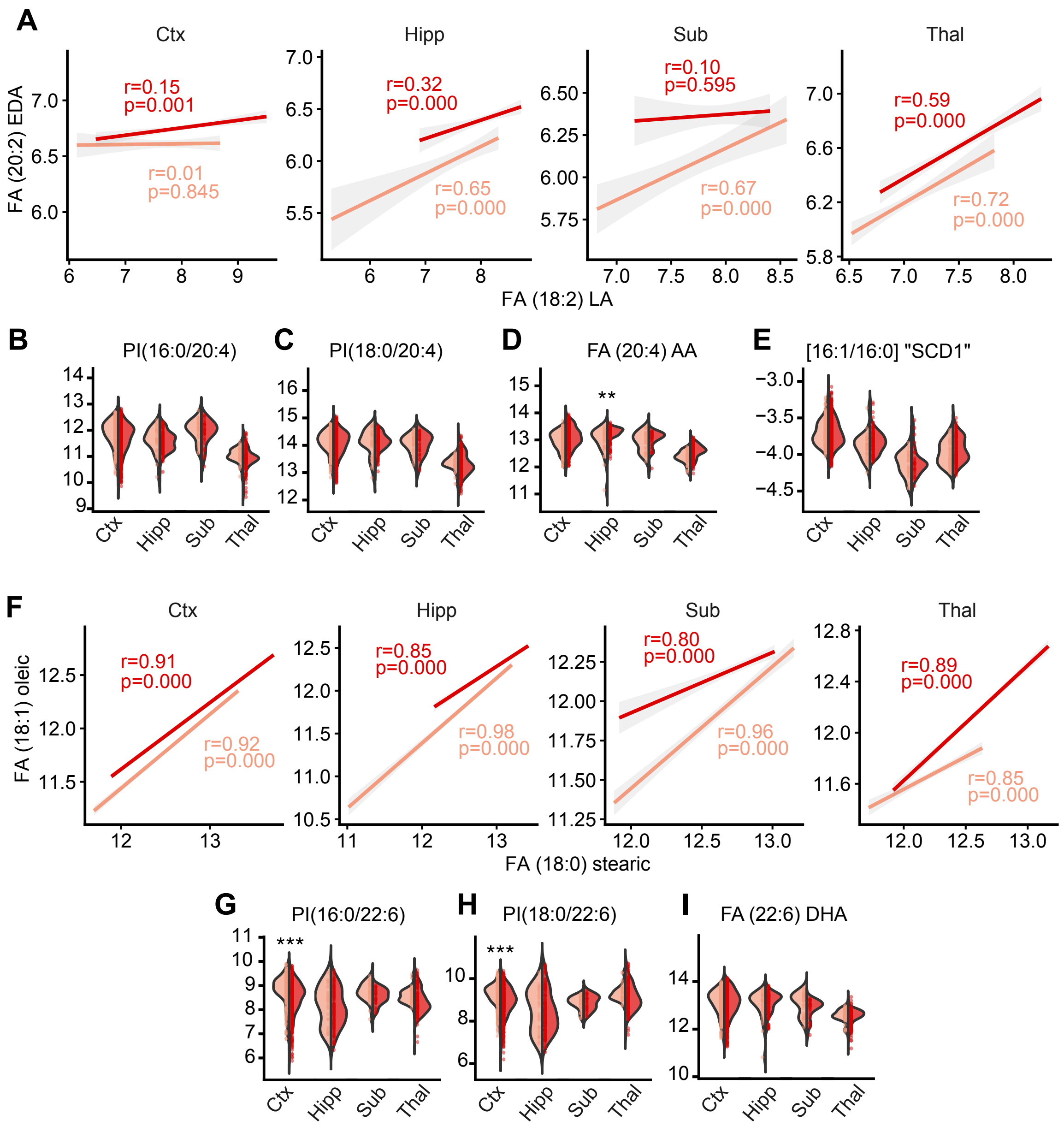

Supplementary Figure 4. Substrate-product correlations and phospholipid controls supporting enzymatic proxy index interpretations. (A) Scatter plots of FA(18:2) LA vs. FA(20:2) EDA across all four regions in young (salmon) and old (red) plaque samples, with Pearson correlation coefficients. EDA/LA coupling is strong in hippocampus, subiculum, and thalamus but absent in cortex at both ages, supporting region-specific suppression of C18 n-6 elongase activity rather than substrate depletion. (B–C) AA-containing phosphatidylinositol species PI(16:0/20:4) and PI(18:0/20:4) show no age-dependent changes in any region, arguing against PLA2-mediated AA mobilisation as a confound. (D) Free AA (FA 20:4) shows no consistent age-dependent depletion and is significantly elevated in hippocampus at old plaque sites, further excluding net phospholipase-driven AA release. (E) The palmitoleic/palmitic ratio [16:1/16:0], a parallel SCD1 substrate pair, is unchanged across all regions, confirming substrate specificity of the SCD1 proxy index. (F) Scatter plots of FA(18:0) stearic vs. FA(18:1) oleic across all four regions in young and old plaque samples. Both age groups show strong coupling ($r > 0.80$) with systematic age-dependent separation in subiculum and thalamus, corroborating elevated proxy for SCD1 Δ9-desaturation activity in those regions. (G–H) DHA-containing phosphatidylinositol species PI(16:0/22:6) and PI(18:0/22:6) are significantly reduced at old plaque sites in cortex, indicating cortex-specific remodelling of DHA-containing PI. (I) Free DHA (FA 22:6) shows no age-dependent changes in any region, dissociating the PI reduction from bulk DHA availability and arguing against PLA2-mediated hydrolysis. All violin plots show young (salmon, left) and old (red, right); comparisons performed at the animal level. $*p < 0.05$, $**p < 0.01$, $***p < 0.001$.

A

Top 10 feature - Age	Importance (Mean decrease Gini)
[ΣPUFA/ΣSFA] "UI"	0.193
[Σ(C20−24)/Σ(C14−18)] "EC"	0.165
[22:6/20:5] "n-3 efficiency"	0.157
[22:5/20:5] "ELOVL2 (I)"	0.139
[20:2/18:2] "ELOVL5 (I)"	0.074
[22:6/22:5] "ACOX1"	0.065
[16:1/16:0] "SCD1"	0.056
[24:5/22:5] "ELOVL2 (II)"	0.056
[20:4/20:3] "FADS1"	0.048
[22:4/20:4] "ELOVL5 (II)"	0.046

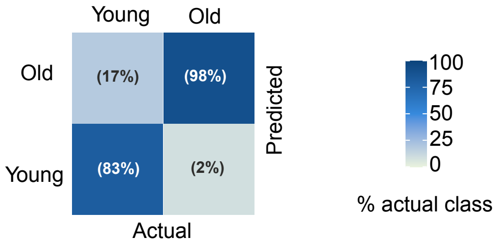

Accuracy: 93.7%

B

Top 10 feature - Region	Importance (Mean decrease Gini)
[16:1/16:0] "SCD1"	0.194
[20:2/18:2] "ELOVL5 (I)"	0.182
[18:1/18:0] "SCD1"	0.128
[22:5/20:5] "ELOVL2 (I)"	0.118
[22:4/20:4] "ELOVL5 (II)"	0.079
[22:6/22:5] "ACOX1"	0.066
[24:6/22:5] "ELOVL2 (III)"	0.064
[24:5/22:5] "ELOVL2 (II)"	0.062
[22:6/22:5] "ACOX1"	0.056
[20:4/20:3] "FADS1"	0.051

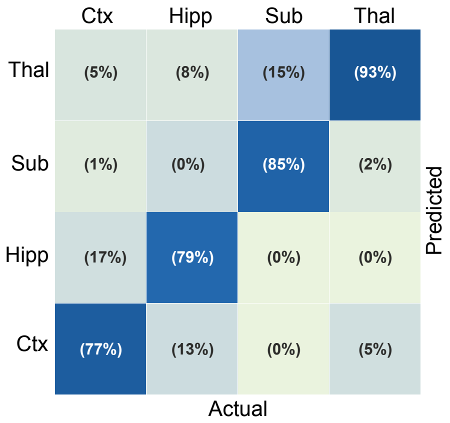

Accuracy: 80.1%

C

Selected top 3 feature - Age	Importance (Mean decrease Gini)
[ΣPUFA/ΣSFA] "UI"	0.341
[Σ(C20−24)/Σ(C14−18)] "EC"	0.336
[22:6/20:5] "n-3 efficiency"	0.323

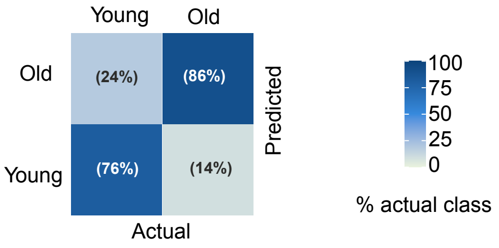

Accuracy: 83%

D

Selected top 4 feature - Region	Importance (Mean decrease Gini)
[16:1/16:0] "SCD1"	0.324
[20:2/18:2] "ELOVL5 (I)"	0.279
[18:1/18:0] "SCD1"	0.206
[22:5/20:5] "ELOVL2 (I)"	0.191

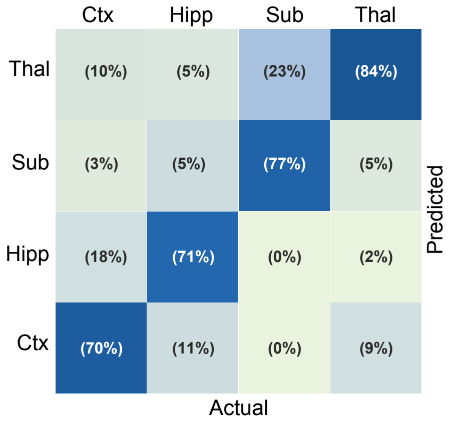

Accuracy: 72.7%

Supplementary Figure 5. Random Forest classification of plaque identity using enzymatic proxy index features (A) Full model age classification: top 10 features ranked by mean decrease in Gini impurity, led by the unsaturation proxy index (UI), elongation capacity proxy index (EC), and n-3 efficiency proxy index, with confusion matrix showing 93.7% overall accuracy. (B) Full model region classification: top 10 features ranked by Gini importance, with SCD1 proxy indices and ELOVL5 I proxy as leading discriminators, and confusion matrix showing 80.1% overall accuracy with strongest performance for subiculum and thalamus. (C) Reduced age model retaining only the top 3 features (UI, EC, n-3 efficiency proxies), achieving 83% accuracy, confirming that the composite indices alone carry the dominant age-discriminating signal. (D) Reduced region model retaining only the top 4 features (proxies for SCD1 [16:1/16:0], ELOVL5 I, SCD1 [18:1/18:0], ELOVL2 I), achieving 72.7% accuracy, demonstrating that a minimal enzymatic index set captures the principal axes of regional plaque lipid identity.

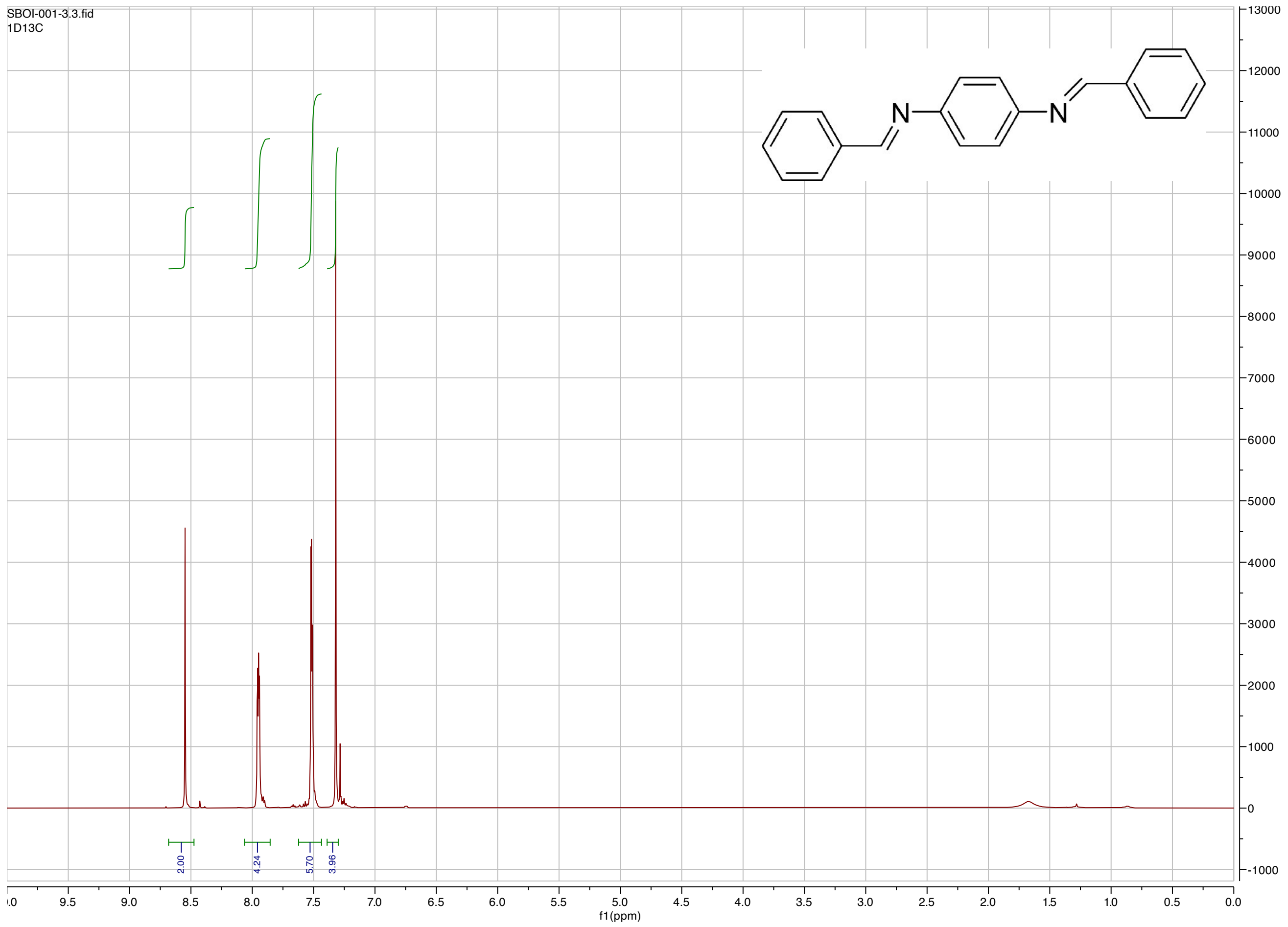

Supplementary Figure 6. NMR characterization of the DBDA matrix. NMR spectrum of DBDA (N,N'-dibenzylidene-1,4-phenylenediamine), the in-house synthesized matrix used for on-tissue free fatty acid detection by MALDI-MSI.

Supplementary Table 1. Annotated features significantly altered in Aβ plaques

m/z	Species
227.201	FA (14:0)
253.217	FA (16:1)
255.233	FA (16:0)
273.186	FA (18:5)
275.201	FA (18:4)
277.217	FA (18:3)
279.233	FA (18:2)
281.249	FA (18:1)
283.264	FA (18:0)
301.217	FA (20:5)
303.233	FA (20:4)
305.248	FA (20:3)
307.263	FA (20:2)
309.280	FA (20:1)
311.295	FA (20:0)
327.232	FA (22:6)
329.248	FA (22:5)
331.264	FA (22:4)
333.279	FA (22:3)
335.295	FA (22:2)
337.311	FA (22:1)
339.327	FA (22:0)
355.264	FA (24:6)
357.278	FA (24:5)
359.296	FA (24:4)
361.310	FA (24:3)
363.326	FA (24:2)
365.342	FA (24:1)
367.357	FA (24:0)
564.536	Cer (d18:1/18:0)
592.567	Cer (d18:1/20:0)
616.470	CerP (d18:1/16:0)

m/z	Species
642.489	HexCer(d18:1/12:0)
726.588	HexCer(d18:1/18:0)
391.226	CPA (16:0)
661.483	DG (40:9)
687.499	DG (42:10)
1091.719	GA2 (d18:1/18:0)
1179.734	GM3 (d18:1/18:0)
1207.768	GM3 (d18:1/20:0)
1235.800	GM3 (d18:1/22:0)
1253.775	GA1 (d18:1/18:0)
1382.818	GM2 (d18:1/18:0)
1410.845	GM2 (d18:1/20:0)
1470.833	GD3 (d18:1/18:0)
1498.866	GD3 (d18:1/20:0)
1516.839	GM1 (d18:1/16:0)
1544.868	GM1 (d18:1/18:0)
1572.904	GM1 (d18:1/20:0)
436.283	LPE (16:0)
480.309	LPE (18:0)
464.313	LPE (P-18:0)
571.289	LPI (16:0)
599.319	LPI (18:0)
619.291	LPI (20:4)
647.465	PA (16:0/16:0)
673.481	PA (16:0/18:1)
718.539	PE (34:0)
738.510	PE (36:4)
722.512	PE (P-16:0/20:4)
750.545	PE (P-18:0/20:4)
857.520	PI (16:0/20:4)
885.551	PI (18:0/20:4)
909.549	PI (18:0/22:6)

m/z	Species
843.538	PI (P-36:3)
865.502	PI (P-38:6)
913.579	PI(18:0/22:4)
786.529	PS (36:2)
806.545	ST (d18:1/18:0)
822.542	ST (d18:1/18:0(2OH))
834.577	ST (d18:1/20:0)
850.572	ST (d18:1/20:0(2OH))
862.607	ST (d18:1/22:0)
878.604	ST (d18:1/22:0(2OH))
888.624	ST (d18:1/24:1)
904.620	ST (d18:1/24:1(2OH))
906.635	ST (d18:1/24:0(2OH))
916.653	ST (d18:1/26:1)
1427.998	CL (70:4)
1425.979	CL (70:5)
1423.963	CL (70:6)
1456.023	CL (72:4)
1454.008	CL (72:5)
1451.995	CL (72:6)
1449.978	CL (72:7)
1447.964	CL (72:8)
1471.966	CL (74:10)
1469.950	CL (74:11)
1473.976	CL (74:9)
1499.990	CL (76:10)
1497.982	CL (76:11)
1495.961	CL (76:12)
1502.009	CL (76:9)
1523.993	CL (78:12)
1521.975	CL (78:13)
